# Physical activity as a causal variable for adolescent resilience levels: A cross-lagged analysis

**DOI:** 10.3389/fpsyg.2023.1095999

**Published:** 2023-02-17

**Authors:** Lingling Guo, Lulu Liang

**Affiliations:** School of Physical Education, Wuhan Sports University, Wuhan, Hubei, China

**Keywords:** physical and mental health, physical activity, adolescent resilience, cross-lagged, adolescent

## Abstract

This study extends research on the relationship between physical activity and adolescent resilience by using cross-lagged analysis. Therefore, it used the Adolescent Resilience Rating Scale and the Physical Activity Scale to conduct a one-year longitudinal follow-up survey on 818 adolescents (50.6% boys), aged 12–17. They completed the questionnaires, providing data on physical activity and adolescent resilience. The results indicated there were significant gender differences in physical activity, but there was no significant gender difference in adolescent resilience; there was a significant positive correlation between physical activity and adolescent resilience; physical activity had a significant predictive effect on adolescent resilience. This study supports the assertion that physical activity has an important impact on adolescent resilience. It further analyzes the possible reasons for this result and contemplates the theoretical and practical significance of the findings, which provides evidence for the study of adolescents’ physical and mental health and enriches the theory of resilience.

## 1. Introduction

Resilience refers to an individual’s ability to adapt to and/or psychologically rebound from adversity, trauma, threats, and stressful events, and can foster environmental adaptability, mental rehabilitation, and emotional relief (Windle, 2011). It can effectively reduce the negative impact of life events on mental health and, thus, represents a positive psychological quality for the healthy development of physical and mental health (Zhou and Hu, 2016; Ma et al., 2022). Based on the importance of resilience, the Chinese government has issued the “Healthy China 2030” Planning Outline, the “Opinions on the Implementation of the Healthy China Action,” and other national documents, which have the clear aims of improving the adolescent population’s ability to address and respond to emergencies and psychological crises. With the continued improvement of China’s productivity, economic development, and material and cultural growth, Chinese teenagers are becoming increasingly prone to lacking resilience and to experiencing trauma, tragedy, threats, or other stressful events (Huang et al., 2019). Concurrently, today’s social environment, in which Chinese adolescents experience pressures associated with parents’ expectations, the urgent need to secure places in further education and/or employment, and the difficulties of maintaining their fast-paced lifestyles, increases the stress they experience. Thus, identifying means of better negotiating the various challenges and discomfort they experience has become increasingly urgent. An insufficient level of resilience in adolescents will lead to delayed mental and social development, and even a lack of independence and responsibility, which can result in game addiction, excessive anxiety, academic pressure, and other symptoms (Zhai and Ji, 2022); this can consequently result in depression, negative coping, suicide, poor academic performance, and other problematic behaviors (Kalisch et al., 2017). In the context of Healthy China 2030, adolescents in middle schools in China are in a phase in which their individual positive psychological qualities are still under formation and, thus, this is a critical period for sound personality construction. Improving the resilience of adolescents in middle school is not only an important way of maintaining their mental health as they get older, but also of improving Chinese society. Thus, it is a prerequisite for the construction of a strong society in a modernist and powerful country.

With the current urgent need to improve the physical and mental health of adolescents, in addition to the implementation of the abovementioned national policies, considerable theoretical and practical research has been conducted in China on this topic. Several of these studies have suggested that active participation in physical activity has positive psychological benefits for adolescents (Guo, 2017). Empirical studies have also shown that, for adolescents, engaging in physical activity can improve negative emotions and transform anxiety, depression, and stress into positive emotions such as happiness, joy, and relaxation (Malm et al., 2019); a possible reason for this effect is that engagement in physical activity contributes to a manifestation of resilience. Similarly, scholars from other countries have conducted related research suggesting that active physical activity participation can promote the psychosocial development of adolescents (Yeh et al., 2016); for example, Fleshner (2005) proposed that regular engagement in moderate physical activity can foster resilience and other improvements and can also buffer stress. Conversely, severe lack of physical activity may cause adolescents to gain weight, and even lead to overweight or obesity (Micheletti Cremasco et al., 2021). Further studies have shown that overweight or obese adolescents have stronger rebellious psychology, concentration and perseverance than normal weight adolescents poor (Hampson et al., 2015). Therefore, adolescents who are not physically active may be more prone to adverse psychological problems. At the same time, adolescents who are severely lacking in physical activity are less able to resist the pressure around them, and are prone to depression, anxiety, loneliness, and decreased happiness (McMahon et al., 2017). They may even appear to be tired of learning, choose to escape from reality, and indulge in the virtual world of the Internet (Liu and Dan, 2018).

When discussing the interactive relationship between behavior and psychology in the context of adolescents’ physical and mental health, academic circles have also advanced the theory that psychological factors such as cognition, emotion, and will may drive the generation of adolescents’ physical behaviors (Ajzen, 1991). In psychological terms, strength represents one’s ability to resist stress, recover, and calm one’s emotions; that is, youth resilience (environmental adaptation ability, mental rehabilitation ability, and emotional-soothing ability) can affect one’s individual physical behavior. When stressful events occur, adolescents with poor environmental adaptability tend to adopt immature defense mechanisms, such as depression, withdrawal, avoidance, and anger, among others (Lee and Choi, 2014). After a crisis event occurs, adolescents with insufficient mental rehabilitation ability are often insufficiently flexible to deal with crisis events, and it is difficult to recover from failure, while their self-confidence is frustrated, and their academic performance deteriorates. In the face of intense pressure, adolescents with insufficient emotional coping skills will have more negative emotions and promote negative thinking patterns. If these emotions are not controlled, they will lead to a vicious circle and aggressive behavior (Alkhawaldeh and Almakanin, 2019). This indicates, that under certain circumstances, the lack of the above three abilities may inhibit the behavior of adolescents. Conversely, from the perspective of positive psychology, individuals with high resilience possess abundant psychological resources, such as life satisfaction, optimism, and serenity and ability to adjust (Pietrzak et al., 2009). When faced with a stressful environment, they can mobilize these resources in a timely manner, cope with difficulties and escape adversity, show good adaptation results, cultivate adolescents’ resilience, and improve the status of physical activities, on the basis of improving adolescents’ social communication skills (Rew and Horner, 2003), which in turn may promote youth physical activity.

**Figure 1:**
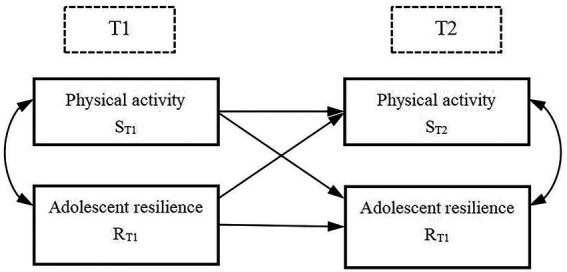
Cross-lagged model.

In summary, there may be a correlation between physical activity and resilience, but for adolescents, does physical activity promote the development of resilience, or does the improvement of resilience affect the behavior of physicals activities? Additionally, from the perspective of social role cognition, will the differences in behavioral expectations and role positioning of men and women in adolescent groups affect youth’s participation in physical activities and resilience performance and reflect gender differences? Therefore, the purpose of this study was to examine the gender heterogeneity of physical activity and adolescent resilience levels through a longitudinal follow-up survey, explore the association between the two using a cross-lagged model, and determine whether physical activity has a significant impact on adolescent resilience. It has a predictive effect (see Figure 1).

## 2. Research hypothesis

### 2.1. Gender differences in physical activity and adolescent resilience

Gender issues are an unavoidable social reality. Many early studies have shown that pervasiveness, persistence, and inheritance of gender stereotypes, are defined as conditioned notions of distinctive characteristics of men and women (Unger and Crawford, 1992). First, the different behavioral expectations and role orientations of men and women, from the perspective of traditional society, lead to certain differences in the individual behaviors and psychological cognitions of men and women, which are naturally reflected in the participation of physicals activities and the performance of adolescent resilience. For a long time, Chinese women have been influenced by the patriarchal ideology of “men are strong and women are weak” and “men are superior to women.” Women often subconsciously obey their self-positioning roles, and they are more responsible for housework, educating children, and caring for the elderly in the family. When compared, men do not have more time to participate in physicals activities. Influenced by Chinese cultural traditions, patriarchal social activities, including the development of various physicals activities, these unconsciously tend to male interests (Hu and Scott, 2016). Secondly, adolescents who are in the period of psychological and physical development, gradually increase their sensitivity to natural gender and may show gender differences in many behavioral habits and social skills. For example, male and female adolescents may have inherent physical differences (Davison, 2004). Thirdly, the emotion attribution theory holds that when experiencing negative experiences, men will complain about the external environment, while women tend to complain about themselves (Sundberg et al., 1991). Moreover, in social interactions, men who are caught in negative situations tend to attribute unpleasant feelings to the outside world’s disapproval, and are more likely to release their emotions; whereas women are more likely to attribute their experiences in negative situations owing to the lack and insufficiency of themselves, as it is easier to close the self and exhibit a lack resilience in self-deprecation. Differences in social role cognition at both the macro and micro levels of adolescents will inevitably have different impacts on participation in physicals activities, and the level of resilience of adolescents. However, there is no specific empirical data to support whether there are gender differences between the two. Based on this, we propose the following two research hypotheses:

*H1*: There are gender differences regarding male and female adolescents’ level of engagement in physical activity.

*H2*: There are gender differences regarding male and female adolescents’ resilience levels.

### 2.2. Relationship between physical activity and adolescent resilience

Researchers have conducted many studies on the resilience of diverse groups of people, different environments, and distinct fields, including “community resilience” (Berkes and Ross, 2013), which emphasizes disaster resilience; “family resilience,” which focuses on how families function to overcome difficulties and resist adversity; “Resilience” (Walsh, 1996); “Social Resilience,” that highlights risk management (Obrist et al., 2010); and “Individual Resilience,” that focuses on promoting the healthy growth of individuals (Ferreira et al., 2020). In the literature on physical activity and adolescents, several scholars in related research, focus on factors affecting adolescent resilience and divide them into extrinsic and intrinsic protective factors, respectively (Gu et al., 2021). For example, external protective factors include family support (Pinkerton and Dolan, 2007), good peer relationships (Peltonen et al., 2014), school teaching adaptation (Guo et al., 2021), and community social group support (Ma et al., 2022); Intrinsic protective factors include strength and trust (Tian, 2015), social competence, self-efficacy, self-confidence, self-awareness and sense of purpose (Zhang and He, 2017), among others. Among the many studies on influencing factors, several scholars pay attention to the role of physical activity in improving the resilience of adolescents (Hu, 2019). For example, in a recent study by Muradyan et al. (2022), it was further shown that regular physical activity can significantly improve individual resilience and well-being Hjemdal et al. (2006) noted that adolescents who participated in physical activity had higher scores on resilience factors. Hegberg and Tone (2015) pointed out that participating in physical activities can increase the psychological resilience of individuals in the face of stress and reduce the incidence of mental health problems. Among them, Ozkara et al. (2016) verified a significant positive relationship between physical activity and resilience. These studies on physical activity have drawn attention to the role of this variable in adolescent resilience. Therefore, urging, encouraging and strengthening adolescents’ active participation in physical activity plays a vital role in promoting adolescent resilience. To examine the potential interaction between physical activity and resilience in adolescents, Moljord et al. (2014) pointed out that the causal relationship between physical activity and adolescent resilience needs to be verified. Based on this, we propose a third hypothesis:

*H3*: There is a causal relationship between physical activity and adolescent resilience.

## 3. Materials and methods

### 3.1. Research subjects

Consequently, in the present study we set adolescents in junior high school and high school as the main test subjects. A cluster sampling method was used to select 865 junior high school and high school students, from four junior high schools and four high schools in Wuhan, Hubei Province, China, respectively. The subjects are young people aged 12–17, and the main test subjects are young people in middle and high school. Data were collected through a questionnaire platform that was implemented in a one-year, two-stage (T_1_ and T_2_) longitudinal follow-up survey format, which measured their levels of resilience (denoted as “R” in the analysis) and engagement in sport (denoted as “S” in the analysis). Overall, 818 participants completed both tests and, thus, comprised the final valid data sample (see Table 1). The first surveys (hereafter referred to as S_T1_, R_T1_) began in September 2020, and 865 questionnaires were initially returned. After reviewing these completed questionnaires in terms of their conformance with “standardized answers,” 818 were deemed valid and their data were retained (effective recovery rate: 94.6%). The second survey (hereafter referred to as S_T2_, R_T2_) began in September 2021, and 865 questionnaires were initially returned; again, 818 responses were deemed valid and their data were retained (effective recovery rate: 92.33%). Thus, the final standard input data was 818 valid data points. Among these, 440 were males and 378 were females, the mean age was 14.040 ± 1.428 years, and 487 were in junior high school, and 331 were in high school (see Table 1). Participants were anonymous and were not paid for the survey; parents/guardians provided required written informed consent.

**Table 1:** Descriptive statistics for physical activity and adolescent resilience.

Test data	Initial (people)	Efficient (people)	Efficient recovery rate (%)	Male adolescents (people)	Female adolescents (people)	Age ± standard deviation
Physical activity	865	818	94.60	440	378	14.040 ± 1.428
S_T1_						
Adolescent resilience						
R_T1_						
Physical activity	886	818	92.33			
S_T2_						
Adolescent resilience						
R_T2_						

### 3.2. Research tools

#### 3.2.1. Physical activity scale

The physical activity rating scale from Kowalski et al.’s (2004) physical activity questionnaire was used in this study. Based on the scoring standard and through localized adaptation, the scale was divided into physical activity frequency, time, and intensity, among others. Each item was scored using a five-point Likert scale. Higher overall scores for the nine items indicated higher levels of physical activity, with ≤19 representing low physical-activity participation, 20–25 representing medium physical-activity participation, and ≥ 26 representing high physical-activity participation; physical-activity participation level was used as an indicator of physical activity.

As shown in Table 2, for S_T1_ and S_T2_ the absolute kurtosis values for each item in the physical activity scale were in the range of 0.396–1.185, the absolute skewness values were in the range of 0.601–0.994, and the minimum standard deviation was 0.483 and 0.516 for S_T1_ and S_T2_, respectively.

**Table 2:** Reliability and validity test analysis indicators of physical activity and adolescent resilience.

Variable		Physical activity	Physical activity	Adolescent resistance	Adolescent resistance
		S_T1_	S_T2_	R_T1_	R_T2_
*N*		10	10	10	10
Validity test (Exploratory factor analysis)	KMO	0.704*	0.743*	0.778*	0.794*
	Bartlett’s test	29.070*	19.212*	21.786*	36.396*
	Cronbach’s alpha	0.807*	0.748*	0.947*	0.978*
Reliability test	Split-half reliability	0.817*	0.694*	0.910*	0.918*
	Stability factor	0.906**		0.769**	
	*df*	8	8	8	8
Minimum standard deviation		0.483	0.516	0.316	0.483
Absolute kurtosis value		0.396–1.185		0.080–1.379	
Absolute skewness value		0.601–0.994		0.132–1.083	

*p < 0.05, **p < 0.01. Kaiser-Meyer-Olkin test.

Physical Activity Scale (S_T1_): The Kolmogorov–Smirnov (KS) test for normal distribution was significant (*p* < 0.05, *df* = 818), regarding exploratory factor analysis, Kaiser-Meyer-Olkin (KMO) = 0.704, and Bartlett’s sphericity test was significant (chi-square = 29.070, *df* = 8, *p* < 0.01). Cronbach’s alpha for the scale was 0.807, and the split-half reliability was 0.817.

Physical Activity Scale (S_T2_): The KS test was significant (*p* < 0.05, *df* = 818), KMO = 0.743, and Bartlett’s sphericity test was significant (chi-square = 19.212, *df* = 8, *p* < 0.01). Cronbach’s alpha was 0.748, and split-half reliability was 0.694.

An initial 10 subjects were retested at 15-day intervals, and the coefficient of stability was 0.906 (*p* < 0.01). Confirmatory factor analysis was then performed; Table 3 shows that, for the physical activity scale at S_T1_, the chi-square degrees of freedom ratio (*χ^2^/df*) was 2.087; meanwhile, for the physical activity scale at S_T2_ the *χ^2^/df* was 3.014.

**Table 3:** Confirmatory factor analysis indicators of physical activity and adolescent resilience.

Variable	Confirmatory factor analysis								
	*χ^2^/df*	*df*	GFI	NFI	IFI	AGFI	CFI	RMSEA	SRMR
Physical activity	2.087	8	0.999	0.984	0.991	0.987	0.991	0.036	0.005
S_T1_									
Physical activity	3.014	8	0.998	0.996	0.998	0.982	0.997	0.050	0.008
S_T2_									
Adolescent resistance	2.657	8	0.991	0.980	0.987	0.977	0.987	0.045	0.023
R_T1_									
Adolescent resistance	2.403	8	0.992	0.987	0.992	0.979	0.992	0.041	0.025
R_T2_									

χ^2^/df, Chi-Square Degrees of Freedom Ratio; AGFI, Adjusted Goodness of Fit Index; CFI, Comparative Fit Index; GFI, Goodness of Fit Index; IFI, Incremental Fit Index; NFI, Normative Fit Index; RMSEA, Root Mean Square Error of Approximation; SRMR, Standardized Mean Square Error.

Combining the results presented in Tables 2, 3 indicates that the Physical Activity Scale had good reliability and validity.

#### 3.2.2. Adolescent resilience rating scale

The Adolescent Resilience Rating Scale was developed based on Liebenberg et al. (2012) Youth Resilience Scale. This scale comprises 28 items. The wording of some items was modified, based on consideration of the subjects’ language reception and comprehension ability; for example, “I know how to express my views in different social environments.” The scale items were all measured using a five-point Likert scale ranging from 1 (“completely inconsistent”) to 5 (“completely consistent),” with total scores indicating the subject’s level of “juvenile resilience.” The higher the overall score for the 28 items, the higher the level of resilience, with ≤79 representing low resilience, 80–119 representing medium resilience, and ≥120 representing high resilience. Resilience level was used as an indicator for youth resilience.

It shown in Table 2, for R_T1_ and R_T2_ the absolute kurtosis values for the Adolescent Resilience Scale were in the range of 0.080–1.379, the absolute skewness values were in the range of 0.132–1.083, and the minimum standard deviation was 0.316 and 0.483 for R_T1_ and R_T2_, respectively.

Adolescent Resilience Scale (R_TI_): The KS test was significant (*p* < 0.05, *df* = 818), KMO = 0.778, and Bartlett’s (Bachlite) sphericity test was significant (chi-square = 21.786, *df* = 8, *p* < 0.05). Cronbach’s alpha was 0.947, and split-half reliability was 0.910.

Adolescent Resilience Scale (R_T2_): The KS was significant (*p* < 0.05, *df* = 818), KMO = 0.794, and Bartlett’s sphericity test was significant (chi-square = 36.396, *df* = 8, *p* < 0.05). Cronbach’s alpha was 0.978, and the split-half reliability was 0.918.

The initial 10 subjects were retested at 15-day intervals, and the coefficient of stability was 0.769 (*p* < 0.01). Confirmatory factor analysis was then performed; Table 3 shows that, for the Adolescent Resilience Scale at R_T1_, *χ^2^/df* was 2.657; meanwhile, for the Adolescent Resilience Scale at R_T2_, *χ^2^/df* was 2.403.

Combining the results in Tables 2, 3 indicates that the Adolescent Resilience Scale had good reliability and validity.

### 3.3. Test process

The research process and methods are all conducted after the moral and ethical review. The research method adopts the questionnaire survey method. First, 10 questionnaires are pre-issued to the subjects, and they are collected after half a month. Simultaneously, the questionnaires are submitted to 5 Chinese experts for review. Reliability and validity analysis of the questionnaire: After the reliability and validity analysis reaches the relevant standard, a large-scale questionnaire is issued to collect data. The distributed questionnaire adopts the online questionnaire completion method. Prior to the questionnaire being distributed, the questionnaire distribution staff informs the subjects of the matters needing attention through face-to-face explanations and timely communication with online social software, explaining the specific requirements for completing the questionnaire, and emphasizing the privacy, confidentiality, and voluntary nature thereof, explaining the purpose and application of the data collected thereby, informing the questionnaire collector of valid information, and letting the subjects understand that they can voluntarily terminate or give up completing the questionnaire at any time. For Chinese teenagers aged 12–15, the class or physical education teachers organize collective use of the school computer classroom to complete the answers, and teenagers aged 16–17 directly distribute online questionnaires to answer. The test taker fills in the answers carefully and checks, then clicks submit to complete the online questionnaire. After the data collection is completed, we organize, cross-check, and archive the questionnaire data.

## 4. Results and analysis

### 4.1. Common method bias test

From the issuance and collection of the questionnaires to the formation of the data-coding processes, structural loss may occur during the detection and selection of data. To reduce the risk of errors, the program control method and the Harman single factor method can be used to test for common method bias.

Program control method: The selected measurement tools were all taken from first-class overseas journals and have been repeatedly proven to have high reliability and validity; the present approach featured voluntary participation, anonymous responses, security and confidentiality measures, and questionnaire answers were provided through digital text.

Harman univariate test: One-way unrotated exploratory factor analysis was performed on all items (except demographic variables), and the results extracted four factors with eigenvalues of >1. The variation rate of the first factor was 25.139% (<40%), thereby proving that the deviation of the actual test data in this study was acceptable.

### 4.2. Hypothesis verification

Descriptive statistics and partial correlation analysis, controlling for gender, showed that S_T1_ and S_T2_ (*r* = 0.747), and R_T1_ and R_T2_ (*r* = 0.850) were all significantly positively correlated (*p* < 0.001) and, concurrently, that S_T1_, R_T1_, R_T2_ (*r* = 0.722, *r* = 0.831), and S_T2_, and R_T1_ and R_T2_ (*r* = 0.777, *r* = 0.837) were also all significantly positively correlated (*p* < 0.001; see Table 4). The above data indicate that physical activity and adolescent resilience have a synchronous and stable correlation across time during one school year, meaning these variables are suitable for a cross-lag analysis.

**Table 4:** Descriptive statistics and results of the partial correlation analysis of the relationship between physical activity and adolescent resilience.

Control variable	Variable	Physical activity	Physical activity	Adolescent resistance	Adolescent resistance
		S_T1_	S_T2_	R_T1_	R_T2_
Gender	Physical activity	1			
	S_T1_				
	Physical activity	0.747***	1		
	S_T2_				
	Adolescent resistance	0.722***	0.777***	1	
	R_T1_				
	Adolescent resistance	0.831***	0.837***	0.850***	1
	R_T2_				
	(M ± SD) _total_	25.19 ± 3.425	25.78 ± 4.676	107.87 ± 18.418	111.60 ± 19.336
	(M ± SD) _Male_	25.97 ± 3.615	27.05 ± 5.173	106.62 ± 16.257	110.81 ± 17.503
	(M ± SD) _Female_	24,28 ± 2.942	24.31 ± 4.676	108.94 ± 20.046	112.29 ± 20.778

***p < 0.001.

#### 4.2.1. Analysis of gender differences in physical activity

Gender-independent sample *t*-tests were performed for S_T1_, and S_T2_ (see Table 5). For both S_T1_ and S_T2_, the F-test for physical activity was significant (*p* < 0.05), rejecting the null hypothesis and indicating heterogeneous data. The independent samples t-test confirmed that there were significant gender differences regarding engagement in physical activity across S_T1_ and S_T2_ (*p* < 0.05), with males showing slightly higher engagement in physical activity levels than females; the difference was statistically significant. Indicating that there were stable gender differences across time regarding level of engagement in physical activity, means therefore, that Hypothesis 1 is verified.

**Table 5:** *T*-test of the relationship between physical activity and adolescent resilience.

Dependent variable	HV-test	Levene’s test		*t*-test				
		*F*	*P*	*T*	*df*	*P*	95% CI	
							LLCI	ULCI
Physical activity	Variance heterogeneity	15.523	0.000***	7.360	813.669	0.000***	1.238	2.138
S_T1_								
Physical activity	Heterogeneity of variance	58.952	0.000***	8.959	774.401	0.000***	2.134	3.332
S_T2_								
Adolescent resistance	Heterogeneity of variance	18.231	0.000***	1.827	813.363	0.068	−0.173	4.812
R_T1_								
Adolescent resistance	Heterogeneity of variance	10.344	0.001***	1.107	815.694	0.268	−1.145	4,109
R_T2_								

***p < 0.001.

#### 4.2.2. Analysis of gender differences in adolescents’ resilience

Gender-independent sample *t*-tests were performed for R_T1_, and R_T2_ (see Table 5). For both R_T1_ and R_T2_, the *F*-test for adolescent resilience was significant (*p* < 0.05), thereby rejecting the null hypothesis and indicating heterogeneous data. The independent samples t-test confirmed that there was no significant gender difference regarding resilience across R_T1_ and R_T2_ (*p* > 0.05), indicating that there was no gender difference in terms of resilience. Therefore, Hypothesis 2 was not verified.

#### 4.2.3. Analysis of the causal relationship between physical activity and adolescent resilience

**Figure 2:**
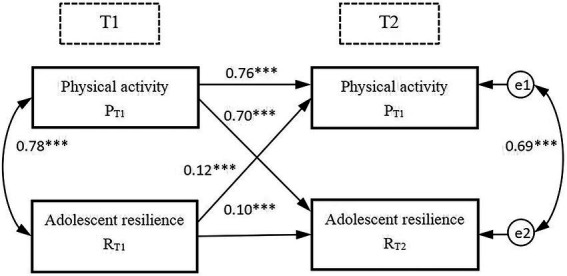
Correlations within the cross-lagged model. ↔ represents the result of the correlation analysis, → represents the result of the regression analysis; the numbers represent the correlation coefficient and unstandardized regression coefficients of the path; in the route of the regression analysis, the solid lines (↔ or →) indicate that the regression coefficient is significant, *** *p* < 0.001.

AMOS was used to build a cross-lag relationship model between physical activity and adolescent resilience, and the maximum likelihood method was used to test the cross-lag effect (Figure 2). The model fit index was *χ^2^/df* = 3.853 (*p* = 0.050, *n* = 818), the goodness of fit index (GFI) was 0.998, and the root mean square error of approximation (RMSEA) was 0.059. The above data show that the constructed model of the cross-lag relationship, between physical activity and youth resilience, had a good degree of fit and adaptability. The causal relationship between physical activity and youth resilience could be investigated using the path coefficient of the structural model. According to Kantowitz et al. (2014) and Eisma et al. (2019), if A_T1_ → A_T2_ correlation>B_T1_ → B_T2_ correlation and, at the same time, A_T1_ → B_T2_ correlation>B_T1_ → A_T2_ correlation, a causal relationship exists between A and B, where A is the cause and B is the effect. As shown in Figure 2, the correlation degree of S_T1_ → R_T2_ (*β* = 0.70, *p* < 0.001) > R_T1_ → S_T2_ (*β* = 0.12, *p* < 0.001). Meanwhile, S_T1_ → S_T2_ correlation (*β* = 0.76, *p* < 0.001) > R_T1_ → R_T2_ correlation (*β* = 0.10, *p* < 0.001). Thus, it is confirmed that there is a relationship between physical activity and youth resilience, with the former influencing the latter; that is, physical activity is the causal variable of adolescent resilience. Hypothesis 3 is verified.

## 5. Discussion

This study measured and analyzed the relationship between physical activity and adolescent resilience based on self-reports of adolescents aged 12–17. The results of the study show that there are significant gender differences in the participation of sports activities, but there is no significant gender difference in the performance of adolescents’ resilience. The results also indicate that there is a causal relationship between physical activity and adolescents’ resilience. Physical activity is the causal variable for the improvement of adolescents’ resilience levels, and the improvement of adolescents’ resilience is the outcome variable of participation in physical activities.

First, there are gender differences in the participation of male and female adolescents in sports activities. An independent sample t-test (Table 5) of the two survey datasets, confirmed that the gender difference for physical activity participation was significant (S_T1,_ S_T2_, *p* < 0.05), and remained stable over time. Male adolescents were, when compared to female adolescents, more active and more frequently engaged in physical activity. Thus, hypothesis 1 (that there is a gender difference in physical-activity participation among adolescents) was verified, and this is consistent with the findings of Dong and Mao (2021), Wang D. et al. (2022), and Wang E. N. et al. (2022).

Accordingly, from the perspective of individual gender cognition, individuals will acquire gender cognition and form gender behavior by imitating adult behavior (Whitfield et al., 2015). As individuals gradually grow up, they are highly susceptible to the gender cognition acquired by early individuals through imitation, so that they will show obvious gender-differentiated behaviors during individual growth (Sukys et al., 2014). Chinese gender concepts have given girls the stereotypical notion of being quiet, so when participating in sports activities, Chinese male adolescents may choose physical activities that show their strength and show off their masculinity; while female adolescents take part in sports activities as well. When choosing, it may be easier to choose low-intensity physical activities with less confrontation and low energy consumption (Leung, 2003).

From the perspective of self-schema theory, early adolescence is a critical period of body image and self-identity, and one of the influencing factors of body image is the media factor in social and cultural factors. “Influenced by female adolescents’ excessive pursuit of slenderness and slender bodies, the result is a decline in their body resistance and system immunity (Tiggemann, 2014). Under the influence of this perception the physical fitness of female adolescents is very likely to differ from that of male adolescents, and physical fitness is the basis of sports activities. Therefore, male and female adolescents may also show certain differences in participation in sports activities. In summary, the cross-time and stable gender differences in adolescent participation in sports activities may be affected by social gender role expectations, individual gender cognition, and social imagery of body expression.

Second, there is no gender difference in the resilience of male and female adolescents. Through the independent sample *T*-test (Table 5) of the two survey data, it can be shown that the level of adolescent resilience has a cross-time and stable gender consistency (R_T1_, R_T2_, *p* > 0.05), therefore hypothesis 2, (that there is a gender difference in the performance of the resilience level of male and female adolescents) has not been verified, which is consistent with the research conclusion of Dong et al. (2019). The distinction and division of labor have resulted in gender differences between male and female adolescents in maintaining interpersonal and emotional relationships. For example, male adolescents prefer to establish friendship and form interpersonal relationships in behavioral interaction experiences, while female adolescents tend to gain peer acceptance through emotional communication and constructing interpersonal relationships (Zhu and Shu, 2022), but with the awakening of gender awareness of relevant stakeholders and the development of society, people gradually recognize stereotypes in gender cognition (Wang and Wang, 2017). Social development and transformation: The development of adolescents’ personalities, the cultivation of psychological quality, and the undifferentiated expectations of their future social roles are evolving rapidly. Therefore, education, family upbringing, and social support serve as educational purposes, nurturing methods, and support methods for adolescent resilience. Considering the specific implementation of the gender-neutral model: After years of specific implementation and operation, the level of resilience of adolescents has also shown that there is no gender difference between men and women.

Finally, there is a causal relationship between physical activity and adolescent resilience. As shown in Figure 2, S_T1_ → S_T2_ correlation (*β* = 0.76, *p* < 0.001) > R_T1_ → R_T2_ correlation (*β* = 0.10, *p* < 0.001); concurrently, S_T1_ → R_T2_ correlation (*β* = 0.70*, p* < 0.001) > R_T1_ → S_T2_ correlation (*β* = 0.12, *p* < 0.001). These results verified hypothesis 3 (that there is a causal relationship between physical activity and adolescent resilience). The unique behavioral characteristics and cluster environment of physical activities affect individuals’ level of emotional regulation and social cognitive ability (Zhang and Qiu, 2019). One’s selection of physical-activity groups can reflect their exercise habits and preferences, while the time spent participating in physicals activities can reflect their level of positive psychological qualities, such as perseverance and self-breakthrough. Based on this, the present study quantified the characteristics of adolescents’ participation in physical activities, by controlling for age and gender through partial correlation analysis, featuring indicators such as physical activity groups and physical activity time. Based on this, a cross-lag relationship model was constructed using the test data for the two stages, and it was confirmed that there was a causal relationship between physical activity and adolescent resilience. This finding was consistent with that of Kamini (2019).

From an ecological perspective, the theory of resilience holds that the formation and development of individual resilience benefits from both the positive support of protective factors and the suppression of negative factors (Chen and Luo, 2012). From this perspective, physical activity, as a protective factor for resilience (Yang et al., 2021), first helps individuals to obtain extremely rich emotional experiences (e.g., through experiencing a pleasant atmosphere, the joy of activity, and emotional excitement), thereby improving their resilience level. Second, physical activities can also help teenagers enhance their individual sense of inner self-worth and external self-control. These important psychological resources, which are composed of related psychological elements, can effectively reduce the negative impact of individual risk factors (Harris et al., 2020); thus, it can be concluded that physical activity can reduce the negative impact of negative factors on adolescents. Combining the two abovementioned positive aspects of physical activity (i.e., a protective factor for adolescents’ resilience and capable of reducing the influence of negative factors on resilience levels), clearly shows that physical activity can effectively improve adolescent resilience.

From the perspective of construction, resilience theory suggests that the formation and development of individual resilience is dependent on one’s mental health, physical function, and social adaptability (Yang, 2014). Existing studies have shown that participating in physical activities can improve an individual’s thinking ability, physical quality, social adaptability, and mental health (Yin and Fu, 2004) and, supporting the above theory, that the comprehensive improvement of these four abilities can effectively promote the formation of resilience. Therefore, this also suggests that, for adolescents, participating in physicals activities is conducive to the formation and development of resilience. Action theory suggests that elevated levels of psychological functioning (resilience) result from the internalization of external actions (physical activity; Hegberg and Tone, 2015), and that adolescents should be encouraged to participate in physical activity to promote resilience. Empirical studies have proven that enhancing and promoting the resilience of adolescents is crucial for their future social growth, study, work, life adaptation, and physical and mental health, among others. Poor resilience may result in poor social adaptability, weak beliefs in survival and life, decreased self-motivation, disorder, and other negative conditions, leading to a series of problems such as depression, suicide, and aggressive behavior (Wang D. et al., 2022; Wang E. N. et al., 2022). Appropriate engagement in physical activity can not only enhance physical fitness, but also promote mental health and alleviate psychological problems such as depression and anxiety (Biddle et al., 2019). Simultaneously, active participation in physical activities may stimulate young people’s awareness of teamwork and help them form good interpersonal relationships, thereby comprehensively improving their interpersonal and communication skills and building good resilience. Thus, through participation in sports activities adolescents can improve their resilience, meaning such participation can not only enhance physical fitness, but also help mental health and, ultimately, secure their healthy development.

## 6. Limitations and implications for future research

The study explored the causal relationship between adolescent sports activities and adolescent resilience through a longitudinal follow-up study across school years and two stages. The results obtained help to reveal the positioning and efficacy of sports activities in developing adolescent resilience, which has certain practical significance, nevertheless, this study still has room for expansion in the future. First, in the analysis of the developmental factors of adolescent resilience in the cross-lag research design, only physical activities were tracked, and other influencing factors were not explored in depth. To ensure the research is more complete, future studies can further explore other factors. Second, this study uses the AMOS statistical application to construct a cross-lag model, to study adolescent physical activity and adolescent resilience. The latent variable R_T2_ → S_T2_ of the model built cannot be directly connected and will be used in future research to be better for the application of statistical methods, such as Mplus statistical modeling software, which builds a more detailed structural equation model. This will ensure the research measurement results will be more accurate. Third, the physical activity scale in this study does not classify specific sports. In the future, we can try to classify and test several types, different items, and distinct intensities of physical activities to more accurately verify and study which type of physical activity offers improvement in resistance to stress and has a more definite impact, and the multi-dimensional division of the physical activity scale makes the research conclusions easier to promote and practical. Fourth, we envisage more extensive international exchanges and cooperation, conduct practical investigations of youth groups in different national conditions, and obtain more extensive and universal research results.

## 7. Conclusion

This study confirms that, among adolescents in middle school, male adolescents participate in physical activity more actively and frequently than female adolescents. However, while there were significant gender differences regarding levels of physical-activity participation, there were no significant gender differences regarding resilience levels. Using a cross-lag research model, empirical analysis confirmed a causal relationship between physical activity and resilience, with participation in physical activity being determined to foster an improvement in resilience levels. These findings suggest that engagement in physical activity is an effective means of building resilience in teenagers; therefore, it has important implications for the physical and mental health of Chinese adolescents. Taken together, it is hoped that the contributions of this study will deepen and further the current limited understanding of the relationship between physical activity and adolescent resilience.

## Data availability statement

The original contributions presented in the study are included in the article/supplementary material, further inquiries can be directed to the corresponding author.

## Ethics statement

Written informed consent was obtained from the individual(s), and minor(s)’ legal guardian/next of kin, for the publication of any potentially identifiable images or data included in this article.

## Author contributions

LG made a significant contribution to the concept of the study and wrote a manuscript. LL collated the experimental data in the manuscript and critically revised the important knowledge content. All authors contributed to the article and approved the submitted version.

## Funding

LG received funding from Humanities and Social Sciences Research Foundation of the Ministry of Education of China (Project No. 20YJA890005).

## Conflict of interest

The authors declare that the research was conducted in the absence of any commercial or financial relationships that could be construed as a potential conflict of interest.

## Publisher’s note

All claims expressed in this article are solely those of the authors and do not necessarily represent those of their affiliated organizations, or those of the publisher, the editors and the reviewers. Any product that may be evaluated in this article, or claim that may be made by its manufacturer, is not guaranteed or endorsed by the publisher.
